# UBX‐390: A Novel Androgen Receptor Degrader for Therapeutic Intervention in Prostate Cancer

**DOI:** 10.1002/advs.202400398

**Published:** 2024-07-03

**Authors:** Soohyun Lee, Hwa‐Ryeon Kim, Yaejin Woo, Jiyoung Kim, Han Wool Kim, Ji Youn Park, Beomseon Suh, Yuri Choi, Jungmin Ahn, Je Ho Ryu, Jae‐Seok Roe, Jaewhan Song, Song Hee Lee

**Affiliations:** ^1^ Ubix Therapeutics Seoul 05836 Republic of Korea; ^2^ Department of Biochemistry, College of Life Science and Biotechnology Yonsei University Seoul 03722 Republic of Korea

**Keywords:** androgen receptor, cancer therapy, degrader, prostate cancer, PROTAC

## Abstract

The androgen receptor (AR) is an attractive target for treating prostate cancer, considering its role in the development and progression of localized and metastatic prostate cancer. The high global mortality burden of prostate cancer, despite medical treatments such as androgen deprivation or AR antagonist therapy, highlights the need to explore alternative strategies. One strategy involves the use of heterobifunctional degraders, also known as proteolysis‐targeting chimeras, which are novel small‐molecule therapeutics that inhibit amplified or mutated targets. Here, the study reports a novel cereblon‐based AR degrader, UBX‐390, and demonstrates its superior activity over established AR degraders, such as ARV‐110 or ARCC‐4, in prostate cancer cells under short‐ and long‐term treatment conditions. UBX‐390 suppresses chromatin binding and gene expression of AR and demonstrates substantial efficacy in the degradation of AR mutants in patients with treatment‐resistant prostate cancer. UBX‐390 is presented as an optimized AR degrader with remarkable potential for treating castration‐resistant prostate cancer.

## Graphical abstract

This study explores the effect of a novel androgen receptor (AR) degrader, UBX‐390. UBX‐390 degrades AR in prostate cancer cells via the ubiquitin‐proteasome system and suppresses AR‐related gene expression, thereby inhibiting the AR signaling pathway. The authors present UBX‐390 as an optimized AR degrader with remarkable anti‐tumor activity in treating castration‐resistant prostate cancer.

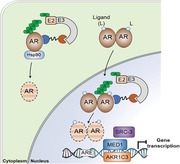

## 1 Introduction

The androgen receptor (AR) is a steroid hormone receptor activated by the male sex hormones, testosterone and dihydrotestosterone (DHT). AR plays a pivotal role in the development and progression of localized and metastatic prostate cancer.^[^ ^1^ ^]^ Despite advancements in medical treatment, prostate cancer remains the second leading cause of cancer‐related deaths among men in the United States and the fifth leading cause of cancer‐related deaths worldwide.^[^ ^2^, ^3^ ^]^ In addition to surgery and radiotherapy, androgen deprivation therapy (ADT) is the first‐line treatment for patients with prostate cancer, to reduce hormone levels in the tumors. Although ADT is effective in most cases, it ultimately leads to metastatic castration‐resistant prostate cancer (CRPC) after 2–3 years.^[^ ^4^, ^5^ ^]^

Another therapy used for prostate cancer is treatment with AR antagonists. The US Food and Drug Administration‐approved first‐generation AR antagonists include flutamide, nilutamide, and bicalutamide.^[^ ^6^, ^7^, ^8^ ^]^ Similar to those administered ADT, patients administered first‐generation AR antagonists acquire resistance, ultimately progressing to CRPC. Therefore, second‐generation AR antagonists with higher AR‐binding affinities and specific targeting of aberrant AR signaling have been developed over the last decade.^[^ ^9^ ^]^ Enzalutamide, a second‐generation AR antagonist, inhibits AR by competing with DHT for binding to AR, and inhibits nuclear translocation, cofactor recruitment, and DNA binding with activated AR.^[^ ^10^, ^11^ ^]^ Although these antagonists contribute to the prolonged survival of patients with CRPC, the condition remains incurable, as patients ultimately acquire resistance to these AR antagonists after long‐term treatment,^[^ ^9^, ^12^ ^]^ through mechanisms such as overexpression of AR proteins.^[^ ^13^ ^]^ Consequently, there is a need for alternative targeted treatment strategies for prostate cancer in patients who are resistant to AR antagonists.

Over the last two decades, heterobifunctional degraders, also known as proteolysis‐targeting chimeras (PROTACs), have been demonstrated to be a novel approach for the discovery and development of small‐molecule therapeutics that induce targeted protein degradation.^[^ ^14^, ^15^, ^16^, ^17^, ^18^ ^]^ A protein degrader is a small molecule that binds to the target protein of interest and is connected to the ligand of an E3 ligase system via a chemical linker.^[^ ^19^ ^]^ Unlike protein inhibitors, these degraders induce protein degradation and offer distinct advantages in targeting proteins amplified in specific diseases, mutated proteins, and chemotherapy‐resistant proteins.^[^ ^20^ ^]^ Protein degraders target drug‐resistant proteins by degrading mutated or overexpressed proteins with relatively low binding affinity.^[^ ^14^ ^]^ Moreover, degraders utilize both the active and allosteric sites of target proteins, which allows the targeting of undruggable proteins, such as transcriptional factors, non‐enzymatic molecules, and structural scaffolding proteins.^[^ ^21^ ^]^

Considering the critical role of AR in CRPC, AR degraders based on the PROTAC concept, designed to eliminate disease‐causing proteins, hold great promise in treating patients with CRPC who display amplified AR expression or resistance to current AR antagonists. ARV‐110, the first‐in‐class AR degrader developed by Arvinas, is currently being evaluated in Phase 2 clinical studies.^[^ ^22^ ^]^ However, the durable effects of AR degradation and whether AR degraders such as ARV‐110 allow for direct dissociation of AR from chromatin are yet to be determined. This study assessed the effects of UBX‐390, a novel cereblon (CRBN)‐based AR degrader, on prostate cancer cells. We compared the effects of UBX‐390 on AR degradation, chromatin binding of AR, and suppression of AR target genes, with those of established AR degraders, under both short‐ and long‐term treatment. Finally, we tested the effect of UBX‐390 on AR mutants found in patients with treatment‐resistant prostate cancer to determine whether it could serve as a novel AR degrader with improved efficacy in treating therapy‐resistant prostate cancer.

## 2 Results

### 2.1 UBX‐390 Potently Degraded AR via the Ubiquitin‐Proteasome System (UPS)

**Figure 1:**
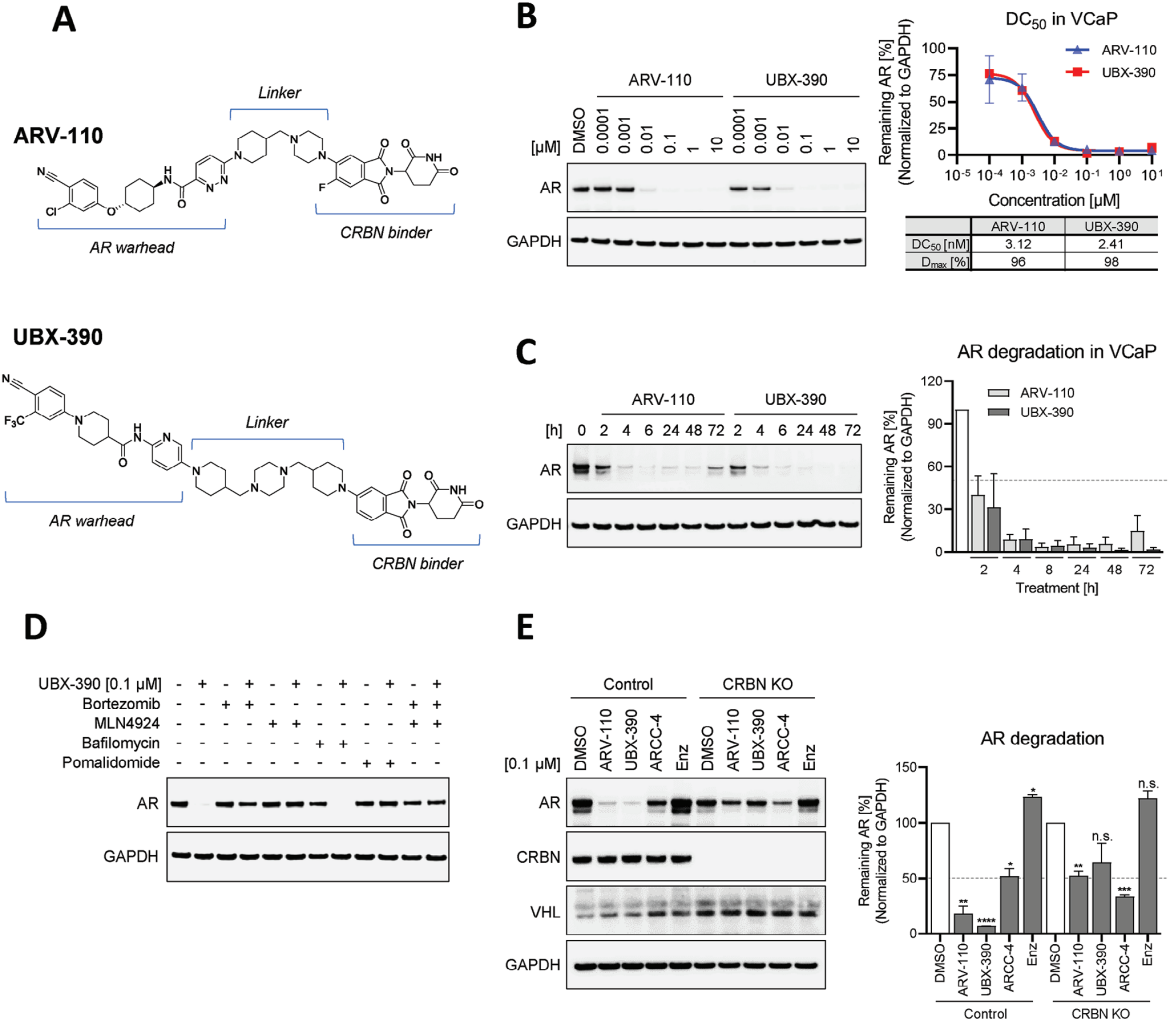
UBX‐390 degrades AR via proteasomal action in a prostate cancer cell line. A) Chemical structures of UBX‐390 and ARV‐110. Immunoblotting results show the AR degradation effect of B) UBX‐390 and ARV‐110 after 20 h of treatment at different doses; and C) 0.1 µm UBX‐390 and ARV‐110 after treatment for different periods. D) Immunoblotting results showing the UPS in VCaP cells. VCaP cells were pre‐treated with 1 µм bortezomib, MLN4924, or bafilomycin, or 10 µm pomalidomide for 1 h and then treated with 0.1 µм UBX‐390 for 4 h. E) The AR degradation effect of 0.1 µm AR degraders, ARV‐110, UBX‐390, and ARCC‐4, and an inhibitor, Enz, in control and CRBN‐knockout CRISPR pool VCaP cell lines. The values for the remaining AR were normalized to those of GAPDH, which served as a loading control. Immunoblotting experiments were conducted independently two times. Data are presented as mean values ± standard error (SE) of the mean. Statistical analyses were performed by Student's *t*‐test using GraphPad PRISM 5. ^*^ *p* < 0.05, ^**^ *p* < 0.01, ^***^ *p* < 0.001, ^****^ *p* < 0.0001, and n.s. means not significant. Enz, enzalutamide; GAPDH, glyceraldehyde‐3‐phosphate dehydrogenase; AR, androgen receptor.

We synthesized a series of AR degrader molecules (*n* = 73) composed of a novel AR warhead that binds to the AR ligand binding domain (LBD) with a dissociation constant (*K*d) of 1.2 nm, thalidomide or lenalidomide moiety as the CRBN E3 ligase binder, and a linker with varied composition to modulate degradation potency and physicochemical properties. Initially, we explored linear linkers of various lengths to establish the appropriate distance between the AR warhead and the CRBN binder, and subsequently optimized the linker with combinations of heterocycles to enhance drug‐likeness. AR degraders were then evaluated for their AR degradation potential by treating the prostate cancer cell line VCaP with 0.01, 0.1, 1, or 10 µm of each compound for 20 h and monitoring the AR protein levels. Among the synthesized AR degraders, UBX‐390, which contains a thalidomide‐based CRBN binder and a piperazine‐based rigid linker, exhibited the strongest AR degradation potency (**Figure** **1**A; Figure S1A, Supporting Information), and was thus selected for further evaluation. To further investigate the AR degradation effect of UBX‐390, we treated VCaP cells with increasing concentrations of UBX‐390 and evaluated the AR levels 20 h post‐treatment. For comparison, we also tested two known selective AR degraders, ARV‐110 and ARCC‐4 (Figure 1A; Figure S1B, Supporting Information). UBX‐390 and ARV‐110 demonstrated substantial AR degradation at concentrations as low as 0.01 µm, showing a half‐maximal degradation concentration (2.41 nm) similar to ARV‐110 (3.12 nm) in VCaP cells (Figure 1B). This effect was consistently observed in 22Rv1 and LNCaP prostate cancer cell lines (Figure S1C, Supporting Information). After observing that these AR degraders induced AR degradation after 20 h of treatment, we treated VCaP cells with UBX‐390, ARV‐110, or ARCC‐4 for different durations up to 72 h without changing the media or adding additional compounds to investigate the kinetics of AR degradation. All degraders showed AR degradation after 2 h of treatment in VCaP cells, and most of the AR was degraded 4 h post‐treatment. Although the AR levels were restored 72 h after treatment with ARV‐110 or ARCC‐4, the effect of AR degradation was sustained for the entire duration of treatment with UBX‐390 (Figure 1C; Figure S1D, Supporting Information). In 22Rv1 and LNCaP cells, a similar pattern was observed for UBX‐390 and ARV‐110, whereas ARCC‐4 showed weaker degradation than the other degraders (Figure S1E, Supporting Information).

Next, we sought to confirm that UBX‐390‐mediated AR degradation depends on the UPS and not on other machinery, such as the autophagy‐lysosomal pathway. For this, we pre‐treated VCaP cells with 1 µm MLN4924 (a neddylation inhibitor), bortezomib (a proteasome inhibitor), bafilomycin (a lysosome inhibitor), or 10 µm pomalidomide (a substrate adaptor for CRBN) before exposure to 0.1 µm UBX‐390 for 4 h. The AR degradation effect was reversed upon pre‐treatment with bortezomib, MLN4924, or an excess amount of pomalidomide, but not bafilomycin. These findings suggest that the AR degradation effect of UBX‐390 is mediated through the proteasome, in a CRBN E3 ligase‐dependent manner (Figure 1D). Since UBX‐390 consists of an AR and CRBN binder, we also tested whether UBX‐390‐mediated AR degradation depends on CRBN recruitment and binding in a CRBN‐knockout (KO) pool VCaP cell line established using clustered regularly interspaced short palindromic repeats (CRISPR) and the CRISPR‐associated protein 9 (Cas9) system. Both the CRBN‐KO and control VCaP cell lines were exposed to 0.1 µm AR degraders or an AR antagonist, enzalutamide for 24 h, and the resulting AR degradation effects were compared. Upon treatment with UBX‐390 and ARV‐110, the control cell line expressing CRBN E3 ligase, displayed significantly stronger AR degradation than the CRBN‐KO cell line. In contrast, ARCC‐4, which comprises an AR binder and a Von Hippel‐Lindau (VHL)‐binding moiety, induced AR degradation in both the CRBN‐KO and control VCaP cell lines (Figure 1E).^[^ ^23^ ^]^ These data indicate that UBX‐390 degrades AR via the UPS and relies on the recruitment of CRBN and its E3 ligase activity.

### 2.2 UBX‐390 Induced Stable Inhibition of the AR Signaling Pathway

**Figure 2:**
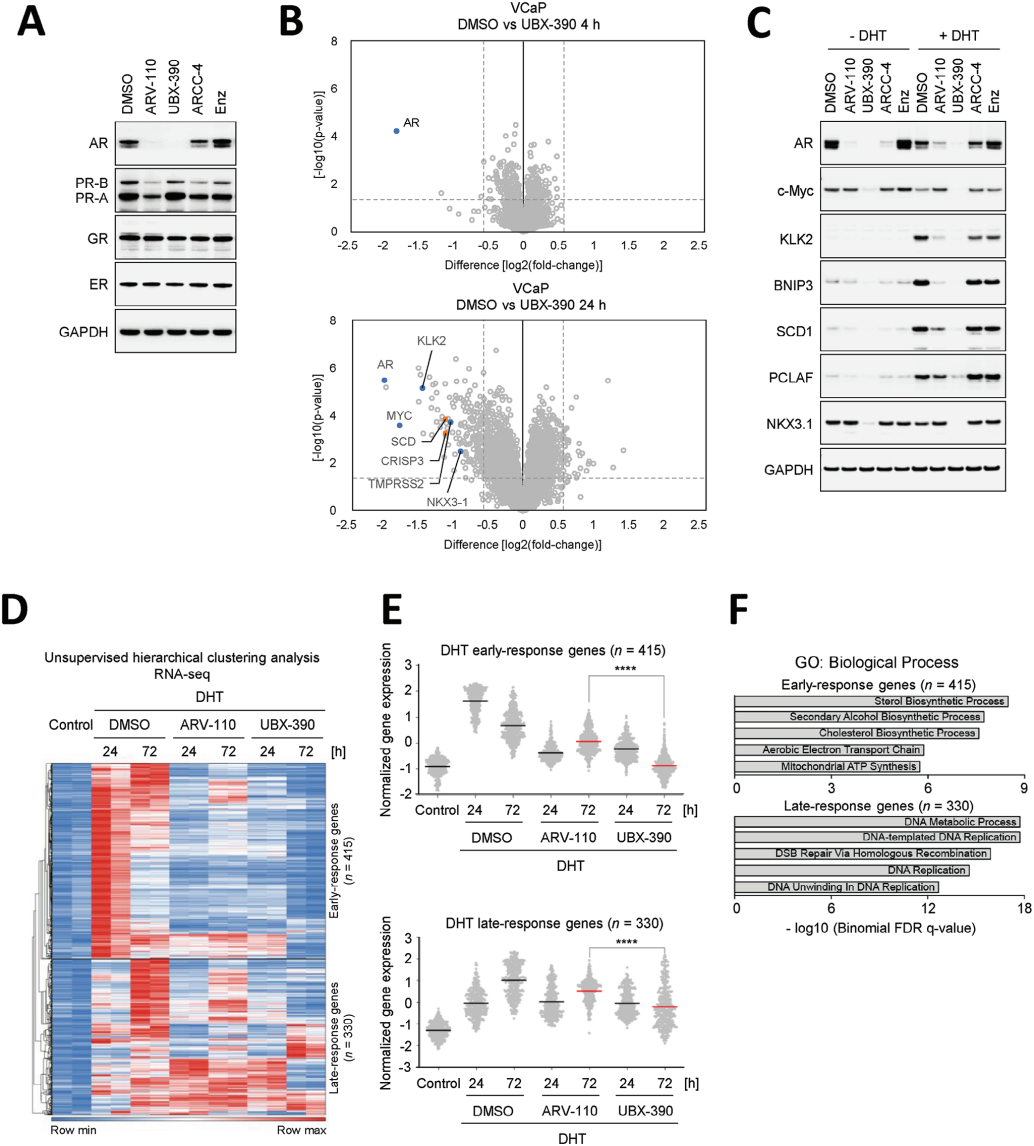
UBX‐390 leads to potent and stable inhibition of the AR pathway. A) Immunoblotting showing the nuclear receptor selectivity of UBX‐390 and other AR degraders in T47D cells at 24 h after treatment with 0.05 µm UBX‐390, ARV‐110, ARCC‐4, or Enz. B) Quantitative proteomic analysis was performed to evaluate proteomic changes in VCaP cells. The cells were treated with 0.1 µm UBX‐390 or DMSO for 4 or 24 h. The lysates obtained were treated with the TMT10plex kit, followed by liquid chromatography‐tandem mass spectrometry‐based proteomic analysis. The volcano plot indicates the protein ranking per abundance ratio (log_2_ fold change) for DMSO and UBX‐390, along with the corresponding statistical *p*‐value. The non‐axial vertical line represents a fold change of ±1.5, whereas the non‐axial horizontal line indicates the threshold of a significant *p*‐value of 0.05. This experiment was performed in triplicate. A total of 7330 proteins were identified in this study. C) Immunoblotting showing the effect of 0.1 µм UBX‐390 or ARV‐110 on AR signaling‐ and prostate cancer‐related proteins in AR‐activated VCaP cells treated with 1 nm DHT. D) Unsupervised hierarchical clustering analysis of DHT‐response genes and E) expression of DHT early‐/late‐response genes in VCaP cells treated with 0.1 µm ARV‐110 or UBX‐390, in combination with 1 nm DHT, for 24 or 72 h. F) Gene Ontology enrichment analysis of DHT early‐response (top, *n* = 415) and late‐response (bottom, *n* = 330) genes. Immunoblotting experiments were conducted independently two times. The proteomics and RNA‐seq experiments were conducted in duplicate. Data are presented as mean values ± SD of the mean. The Reads Per Kilobase per Million mapped reads (RPKM) values were subsequently normalized using Z‐scores. Statistical analyses were performed by Student's *t*‐test using GraphPad PRISM 5. ^****^ *p* < 0.0001. DHT, dihydrotestosterone; Enz, enzalutamide; AR, androgen receptor.

To assess whether the degradation effect of UBX‐390 is selective for AR, we utilized the T47D cell line, which expresses four different steroid hormone receptors: AR, glucocorticoid receptor (GR), estrogen receptor (ER), and progesterone receptor (PR).^[^ ^24^ ^]^ All AR degraders, including ARV‐110, UBX‐390, and ARCC‐4, reduced AR levels without altering GR and ER levels. However, unlike UBX‐390, treatment with ARV‐110 or ARCC‐4 lowered the PR levels. This observation confirmed that UBX‐390 selectively degraded AR while sparing the degradation of other related nuclear receptors (**Figure** **2**A).

To assess the off‐target effects of UBX‐390 and further investigate its potential inhibitory effect on the AR pathway, we conducted a quantitative proteomic analysis using VCaP cells treated with UBX‐390 for 4 or 24 h. This revealed the selective degradation of AR by UBX‐390 among a pool of 7330 proteins, since AR emerged as most substantially downregulated following a 4 h treatment with UBX‐390. In contrast, after 24 h of treatment with UBX‐390, the levels of proteins associated with the AR and prostate cancer signaling pathways were downregulated compared to those observed after treatment with dimethyl sulfoxide (DMSO) (Figure 2B). To validate this, we conducted western blot analyses in VCaP cells treated with 1 µm UBX‐390 for 4, 24, or 72 h and found a decrease in the levels of proteins related to the AR pathway and prostate cancer biology (*i.e*., Myc and NK3 homeobox 1 (NKX3.1)) over time following treatment (Figure S2, Supporting Information). To investigate whether this pattern persisted under activated AR signaling with DHT, western blotting was performed using samples from VCaP cells treated with 0.1 µm UBX‐390 for 72 h. In the presence of DHT, there was drastic AR degradation by UBX‐390 compared to that by ARV‐110. Moreover, in the presence of DHT, UBX‐390 treatment significantly reduced the increased levels of AR signaling‐ and prostate cancer‐related proteins, as compared to ARV‐110 treatment (Figure 2C). These findings suggest that UBX‐390 selectively and functionally inhibits the AR signaling pathway.

Next, we conducted an RNA sequencing (RNA‐seq) analysis to determine whether the degradation of the AR protein by UBX‐390 led to the inhibition of gene expression in the AR signaling pathway. Using unsupervised hierarchical clustering, we identified 415 early‐response genes that responded rapidly to DHT treatment and displayed increased expression within 24 h. In addition to this, 330 late‐response genes showed their highest expression levels at 72 h (Figure 2D). The expression of early‐response genes was effectively suppressed upon treatment with ARV‐110 and UBX‐390 for 24 h. However, the expression of early‐response genes was further suppressed after treatment with UBX‐390 for 72 h but was slightly restored after treatment with ARV‐110 for 72 h (Figure 2E). This was likely due to the restoration of the AR protein after treatment with ARV‐110 for 72 h, as confirmed in Figure 1C. Similarly, the expression of late‐response genes to DHT was more effectively inhibited by UBX‐390 than by ARV‐110 as UBX‐390 sustained the effect for 72 h. Notably, the top five Gene Ontology (GO) terms for DHT early‐response genes included sterol and cholesterol biosynthesis. In contrast, those for late‐response genes were related to DNA replication and repair processes (Figure 2F), suggesting a potential and distinct contribution of UBX‐390 to prostate cancer biology.

Moreover, we observed a significant decrease in the number of genes upregulated and downregulated by ARV‐110 (by at least two‐fold) 72 h post‐treatment, indicating that the effect of ARV‐110 could not be sustained for 72 h. In contrast, the number of genes upregulated by UBX‐390 remained stable during this period, while the number of downregulated genes increased compared to 24 h post‐treatment, confirming that UBX‐390 exerts a prolonged effect on AR degradation for 72 h (Figure S3A,B, Supporting Information). Consistent with these findings, androgen‐response signature genes were consistently and effectively suppressed by UBX‐390 for up to 72 h, whereas ARV‐110 did not show this sustained suppressive effect (Figure S3C, Supporting Information). Given the established role of the PI3K‐AKT‐mTOR signaling pathway in prostate cancer development, progression, and resistance to therapy, we examined the expression patterns of genes involved in this pathway.^[^ ^25^ ^]^ Treatment with ARV‐110 and UBX‐390 for 72 h revealed that UBX‐390 exerted a more potent suppressive effect on mTOR pathway genes compared to ARV‐110, highlighting its enhanced anti‐cancer efficacy (Figure S3D, Supporting Information).

### 2.3 UBX‐390 Acted on Nuclear AR for Degradation

**Figure 3:**
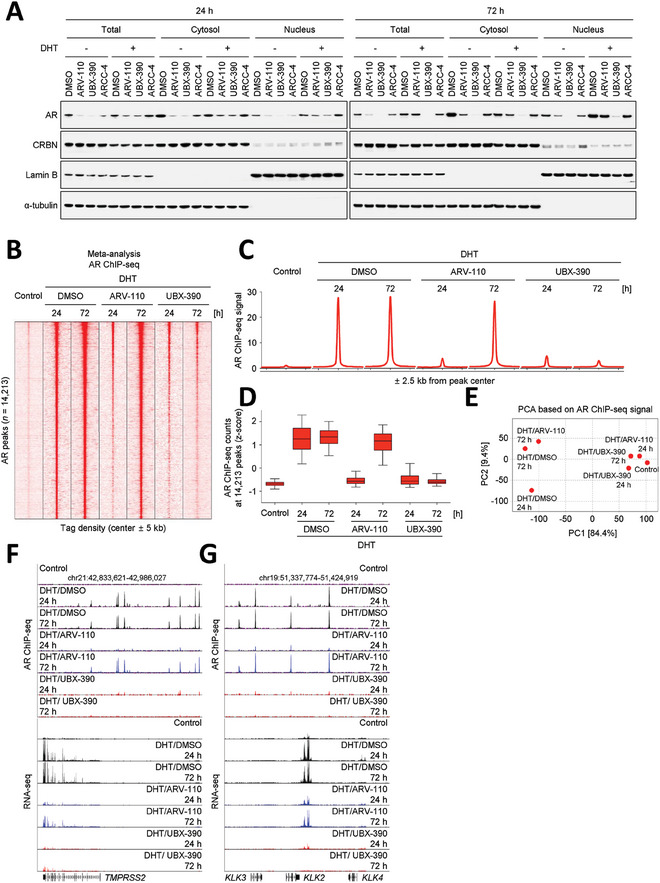
UBX‐390 interferes with DHT‐dependent AR recruitment on the chromatin. A) Assessment of the AR degradation effect of 0.1 µm AR degraders in the cytosol and nucleus after treatment with 1 nm DHT for 24 and 72 h, followed by nuclear fractionation. B) Density plot of AR ChIP‐seq signals in 0.1 µm AR degrader‐treated VCaP cells at the indicated genomic regions (10 kb around the center of the AR peak, *n* = 14213). C) Metagene representations based on AR ChIP‐seq of AR‐occupied regions in the indicated cells. D) Box plot of AR ChIP‐seq counts at the AR peak (*n* = 14213). E) Principal component analysis based on AR ChIP‐seq signals. F,G) Representative browser tracks of AR ChIP‐seq and RNA‐seq in the indicated cells at the F) *TMPRSS2* and G) *KLK2/3/4* loci. Immunoblotting experiments were conducted independently two times. AR ChIP‐seq and RNA‐seq experiments were conducted in duplicate. Data are presented as mean values ± standard deviation (SD) of the mean. AR ChIP‐seq counts were subsequently normalized using Z‐scores. DHT, dihydrotestosterone; Enz, enzalutamide; GAPDH, glyceraldehyde‐3‐phosphate dehydrogenase; AR, androgen receptor.

To investigate the localization of AR degradation induced by UBX‐390, we treated VCaP cells with UBX‐390 for 24 and 72 h in the presence of DHT, followed by nuclear fractionation. Successful completion of the nuclear fractionation process was verified by the detection of lamin B and α‐tubulin, which are representative of the nuclear and cytosolic fractions, respectively. We observed that CRBN was present in both the cytosol and nucleus, enabling UBX‐390 to induce the degradation of both nuclear and cytosolic AR. After DHT treatment, the AR moved to the nucleus within 24 h, and this effect was stronger after 72 h. The degradation of AR was observed in both the cytosol and nucleus of VCaP cells at 24 h post‐treatment with 0.1 µм AR degraders (ARV‐110 and UBX‐390) in the presence of DHT, despite the lower level of nuclear CRBN than cytosolic CRBN. The AR degradation effect of ARCC‐4 was weaker than that of the other AR degraders (ARV‐110 and UBX‐390) in both the cytosol and nucleus of VCaP cells at 72 h post‐treatment. The degradation effect of UBX‐390 on the AR persisted for 72 h, whereas the AR expression level increased with ARV‐110 treatment after 72 h (**Figure** **3**A). This effect was confirmed by immunofluorescence (Figure S4, Supporting Information). Nuclear fractionation and immunofluorescence analyses suggested that UBX‐390 is a more potent and effective AR degrader than ARV‐110.

Subsequently, we performed chromatin immunoprecipitation sequencing (ChIP‐seq) using AR antibodies to confirm whether AR degraders suppress AR chromatin occupancy. Upon induction of AR translocation to the nucleus by DHT treatment, the heightened chromatin binding of AR was effectively suppressed by both ARV‐110 and UBX‐390 (Figure 3B). Consistent with the earlier RNA‐seq results, UBX‐390 significantly inhibited chromatin binding following 24 and 72 h treatments. Surprisingly, most AR were re‐associated with chromatin upon ARV‐110 treatment for 72 h (Figure 3C,D), which could be attributed to the restoration of AR proteins in the nucleus after ARV‐110 treatment for 72 h (Figure 3A). Consistently, AR ChIP‐seq signal‐based principal component analysis revealed that AR chromatin occupancy differed between UBX‐390‐ and ARV‐110‐treated VCaP cells, after 72‐h of treatment conditions (Figure 3E). In response to DHT treatment, AR binding to genomic regions related to the AR signaling pathway was severely reduced by UBX‐390 treatment, which likely inhibited gene expression (Figure 3F,G). Taken together, the chromatin‐oriented evaluation of AR degraders strongly supports the prolonged and enhanced efficacy of UBX‐390 in inhibiting the AR signaling pathway.

### 2.4 UBX‐390 Demonstrated Therapeutic Effects in Prostate Cancer

**Figure 4:**
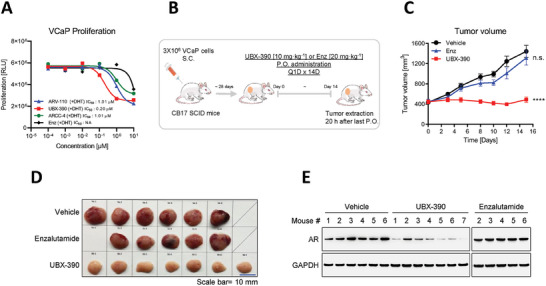
UBX‐390 exhibits therapeutic activity in xenograft models of prostate cancer. A) Evaluation of the inhibitory effects of ARV‐110, UBX‐390, ARCC‐4, and enzalutamide on cell proliferation of VCaP cells, 6 d post‐treatment. This experiment was performed in duplicate. B) Experimental scheme of the in vivo experimental design of the VCaP xenograft model. (*n* = 7 for each group) C) Graph showing tumor growth following vehicle, UBX‐390, or enzalutamide administration to the VCaP xenograft mouse models. D) Representative photographs of subcutaneous tumors derived from the VCaP cells. Scale bar = 10 mm. E) Immunoblotting showing the AR degradation effect of UBX‐390 in tumor tissues extracted from the xenograft model. The proliferation experiments were conducted in duplicate. Data for tumor growth are presented as mean values ± SE of the mean. Statistical analyses were performed by one‐way analysis of variance (ANOVA) tests using GraphPad PRISM 5. ^****^ *p* < 0.0001, and n.s. means not significant.

To explore the biological consequences of UBX‐390‐mediated AR degradation, we investigated its anti‐tumorigenic activity in vitro and in vivo. While all the three tested AR degraders displayed anti‐proliferative effects in VCaP cells, UBX‐390 demonstrated a lower half‐maximal inhibitory concentration value (0.2 µм) than ARV‐110 and ARCC‐4 (both 1.01 µm) (**Figure** **4**A). Moreover, we performed lactate dehydrogenase (LDH) cell cytotoxicity assays in HepG2 cells and human peripheral blood mononuclear cells (PBMCs) to determine the in vitro cytotoxic effects of UBX‐390. These cell lines were chosen to ensure that UBX‐390 does not exhibit cytotoxic effects in liver or blood cells, which could lead to potential side effects. The results demonstrated no significant cytotoxicity at various concentrations 24 h post‐treatment (Figure S5A,B, Supporting Information).

We further investigated the therapeutic effects of UBX‐390 in a mouse xenograft model of prostate cancer generated by subcutaneously injecting VCaP cells near the scapulae of 6‐week‐old CB17/SCID mice using Matrigel. Oral administration of the vehicle, enzalutamide (20 mg kg^−1^), or UBX‐390 (10 mg kg^−1^) was initiated when the tumors reached a volume of 400–450 mm^3^ and continued for 14 d (Figure 4B). No loss in body weight was observed in mice treated with enzalutamide or UBX‐390 (Figure S5C, Supporting Information). UBX‐390 completely inhibited tumor growth, whereas enzalutamide did not significantly inhibit tumor growth, as compared to the vehicle, after once‐daily oral administration for 14 d (Figure 4C,D). Additionally, western blotting confirmed that UBX‐390 administration significantly induced AR protein degradation in the tumor tissues of the VCaP xenograft model, thus indicating that the tumor growth inhibitory effect of UBX‐390 was due to AR degradation (Figure 4E) and demonstrating the anti‐tumor activity of UBX‐390 in vivo (Figure 4C,D).

### 2.5 Broad Activity of UBX‐390 on Therapy‐Resistant AR Mutations

**Figure 5:**
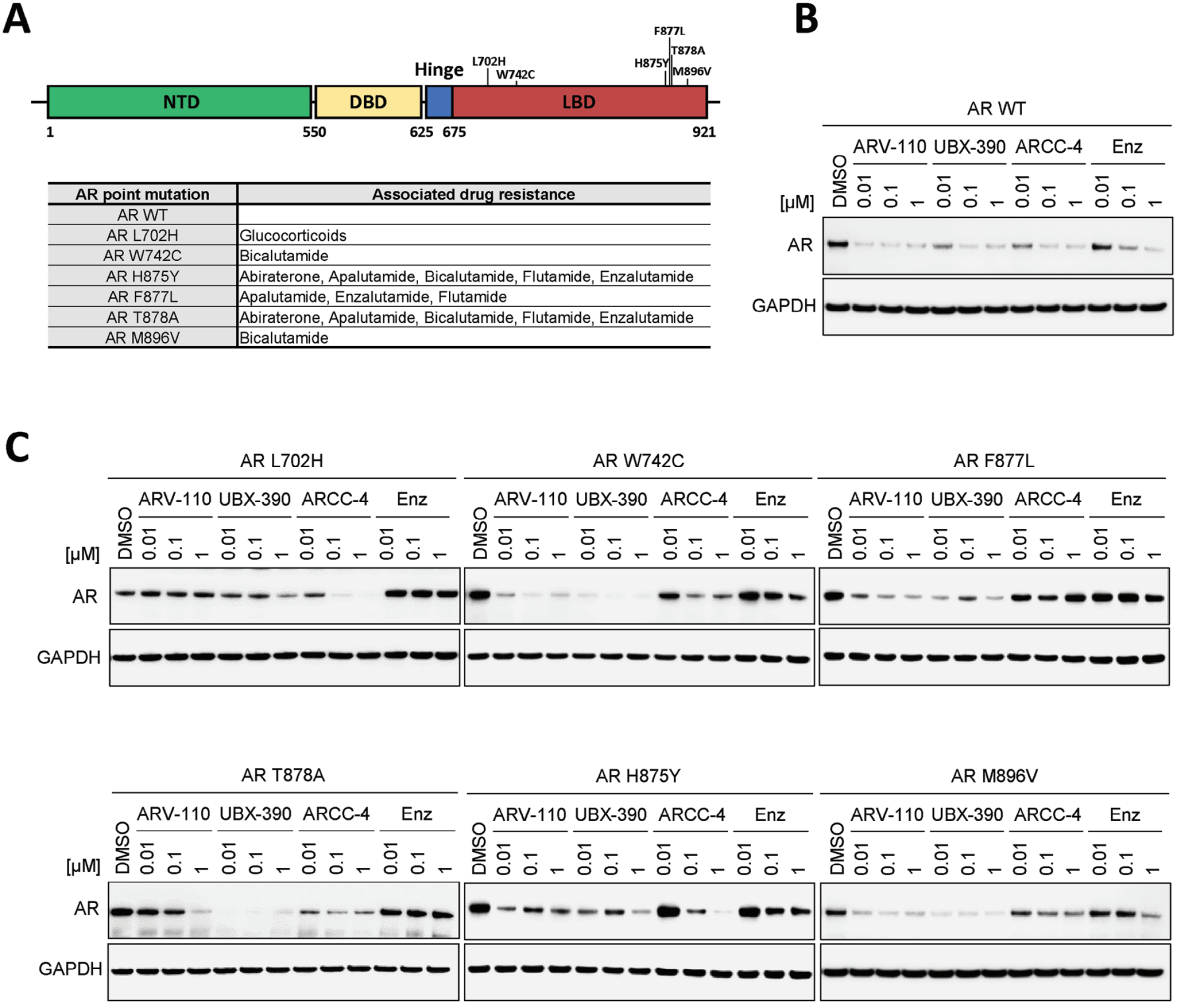
UBX‐390 showed broad activity against therapy‐resistant AR mutations in the ligand‐binding domain. A) Structure of AR and its activating point mutations. Immunoblotting showing the AR degradation effect of AR degraders, ARV‐110, UBX‐390, and ARCC‐4, and an AR antagonist, enzalutamide, in HEK293 cells with transient expression of B) WT AR or C) various AR mutants, after 24 h of treatment. WT, wild‐type; Enz, enzalutamide; GAPDH, glyceraldehyde‐3‐phosphate dehydrogenase; AR, androgen receptor.

Currently used AR antagonists, including hydroxyflutamide, bicalutamide, and enzalutamide, target the androgen‐binding site of the receptor and compete with endogenous testosterone or DHT.^[^ ^26^ ^]^ However, AR mutations at this ligand‐binding site are associated with poor prognosis and resistance to conventional prostate cancer treatments.^[^ ^26^ ^]^ These spontaneous mutations often arise in patients after long‐term treatment with drugs indicated in **Figure** **5**A, leading to expanded binding specificity for AR and an increase in AR signaling.^[^ ^27^ ^]^

To investigate the degradation activity of UBX‐390 against these drug‐induced AR mutants, expression plasmids for diverse mutants, such as L702H, W742C, H875Y, F877L, T878A, and M896V, were transiently transfected into HEK293 cells. The protein levels of each mutant were monitored after 24 h of treatment with UBX‐390, ARV‐110, ARCC‐4, or enzalutamide, at concentrations of 0.01, 0.1, and 1 µm. Enzalutamide was used as the negative control. All three AR degraders (ARV‐110, UBX‐390, and ARCC‐4) strongly degraded wild‐type AR in HEK293 cells (Figure 5B). While UBX‐390 exhibited the most potent degradation effect on AR T878A and H875Y, both UBX‐390 and ARV‐110 showed stronger degradation of AR M896V, F877L, and W742C than ARCC‐4 (Figure 5C). UBX‐390 and ARV‐110 did not induce degradation in cells transfected with the expression plasmid for AR L702H (Figure 5C).^[^ ^28^ ^]^ UBX‐390 degraded the AR mutants most effectively, suggesting that it is a promising therapeutic option over other AR degraders or inhibitors for treating patients with drug‐resistant prostate cancers harboring AR mutations.

We also examined whether UBX‐390 could induce degradation of the splice variant, AR‐V7, in VCaP cells. AR‐V7 is a well‐known AR splice variant in prostate cancer characterized by the absence of the C‐terminal ligand‐binding domain while retaining the N‐terminal transcriptional elements that activate AR signaling independently of ligands.^[^ ^29^ ^]^ As expected, given that UBX‐390 targets the AR ligand‐binding domain, UBX‐390 could not directly induce degradation of AR‐V7 24 h post‐treatment in VCaP cells, a CRPC cell line expressing the splice variant, AR‐V7.^[^ ^30^ ^]^ Therefore, this result confirms that UBX‐390 specifically targets the ligand binding site of AR (Figure S6A, Supporting Information).

Furthermore, UBX‐390 displayed a stronger anti‐proliferative effect than ARV‐110 in VCaP, LNCaP cells (T878A mutation, no splice variant) and 22Rv1 (H875Y mutation, expresses AR variants such as AR‐V7, ARV‐12, and AR‐V1), whereas neither UBX‐390 nor ARV‐110 had any effect on DU145 cells, which is known as an AR‐negative cell line.^[^ ^31^ ^]^ This suggests that UBX‐390 is effective in AR‐positive cell lines, regardless of the AR mutation (Figure S6B,C, Supporting Information).

## 3 Discussion

The AR signaling axis plays a key role in the progression of prostate cancer, including in the lethal chemotherapy‐resistant form of CRPC that arises from ADT failure. Although first‐line treatment with ADT and second‐generation AR antagonists, such as enzalutamide and abiraterone, are used to inhibit AR signaling in patients with prostate cancer, AR activity persists due to direct alterations to the signaling axis.^[^ ^32^ ^]^ Therefore, the use of AR degraders for targeted protein degradation has significant potential as a crucial therapeutic approach to address the needs of patients with CRPC. This study provides meaningful insights into the development of this therapeutic domain.

The AR degrader tested in this study, UBX‐390, exhibited a more potent and prolonged effect on AR degradation than ARV‐110, which failed to sustain its AR degradation effect for 72 h. Moreover, proteomics data showed that UBX‐390 reduced the levels of proteins, such as Kallikrein‐2 (KLK2) and NKX3.1, which are related to the AR signaling pathway. This result was consistent with the RNA‐seq data, which showed that UBX‐390 reduced the expression of genes that were robustly upregulated by DHT. DHT induced the upregulation of genes at 24 h (early‐response genes) and 72 h (late‐response genes) post‐treatment. Early‐response gene expression was suppressed after 24 h of treatment with UBX‐390, and the effect was prolonged for 72 h. In contrast, late‐response gene expression was suppressed after 72 h of treatment. ARV‐110 also suppressed early‐response gene expression after 24 h of treatment; however, this effect was not sustained, and the expression of these genes increased again after 72 h. Moreover, UBX‐390 induced down‐regulation of androgen‐response and mTOR signaling‐related genes and sustained this effect for 72 h. The PI3K‐AKT‐mTOR signaling pathway is well known in prostate cancer for its critical role in tumor formation, cancer progression and drug resistance.^[^ ^25^ ^]^ This potent prolonged inhibition of the AR response and mTOR signaling pathway by UBX‐390 explains its more potent anti‐proliferative and tumor growth inhibitory effects compared to ARV‐110.

UBX‐390 is composed of a ligand that binds to the AR and is tethered with another ligand that binds to CRBN E3 ligase. CRBN and VHL are E3 ligases that are the most widely used substrates for heterobifunctional degraders to induce the ubiquitination and proteasomal degradation of proteins of interest, because they are ubiquitously expressed in cancer cells.^[^ ^33^ ^]^ Treatment with UBX‐390 induced AR degradation in both the cytosol and nucleus of VCaP cells, suggesting that UBX‐390 can recruit AR to CRBN in the nucleus, even when CRBN is less abundant in the nucleus than in the cytosol.

Several mechanisms may underlie drug resistance in patients with CRPC. Recent studies have indicated that the AR is more localized in the nucleus of CRPC cells than in those of hormone‐sensitive cells. Nuclear AR are not exported to the cytosol, whereas cytosolic AR are imported into the nucleus during androgen deprivation.^[^ ^27^, ^34^ ^]^ AR degradation mostly occurs in the nucleus, and a decreased rate of nuclear degradation leads to increased nuclear AR stability in CRPC. AR acts as a transcription factor in the nucleus and increased nuclear AR levels may be one of the reasons for drug resistance in CRPC.^[^ ^27^ ^]^ We showed that UBX‐390 induced strong AR degradation in both the cytosol and nucleus of VCaP cells in the presence of DHT. Therefore, UBX‐390 is a potential therapeutic option for treating patients with CRPC that is resistant to therapy. These findings suggest that degraders with CRBN binders can be broadly used to treat different types of diseases if the key disease‐driving protein is present in the nucleus and/or cytosol.

Another mechanism that leads to resistance to ADT or AR antagonists in patients with CRPC is increased AR signaling due to AR protein overexpression,^[^ ^13^ ^]^ AR gene amplification,^[^ ^35^ ^]^ AR gene mutations,^[^ ^36^ ^]^ or AR splice variant expression.^[^ ^37^, ^38^ ^]^ Spontaneous mutations, that occur in patients after long‐term drug treatment (Figure 5A), lead to expanded AR‐binding specificity and increased AR signaling.^[^ ^27^ ^]^ Protein degraders could be the best option for targeting drug‐resistant proteins, as they are able to induce the degradation of mutated or overexpressed proteins, even with relatively low binding affinity.^[^ ^14^ ^]^ We showed that the AR degrader, UBX‐390, induced the degradation of AR antagonist‐derived AR mutants, suggesting its potential for treating drug‐resistant prostate cancers with AR mutations.

We propose our AR degrader, UBX‐390, as an advancement over ARV‐110 in scientific and therapeutic efficacy. Treatment with UBX‐390 led to a significant reduction in AR binding to genomic regions associated with the AR signaling pathway. This resulted in suppressed gene expression, elucidated through RNA‐sequencing analysis of the transcriptome. Moreover, to date, there has been no prior assessment of the on‐target effects of ARV‐110, a compound currently in clinical stages, using ChIP‐seq analysis. In this study, ChIP‐sequencing analysis was conducted to reveal alterations in AR‐chromatin binding for both ARV‐110 and the newly introduced UBX‐390. This methodological approach represents a significant scientific advancement, providing crucial insights for therapeutics strategies in patients with mCRPC. Our findings demonstrated that UBX‐390 effectively inhibited AR chromatin binding for 72 h, whereas re‐association of AR with chromatin occured with ARV‐110 treatment within the same timeframe, potentially due to the restoration of AR proteins in the nucleus. This sustained effect of UBX‐390 underscores its potential as an enhancement over ARV‐110.

## 4 Conclusion

Our study identified UBX‐390 as a potent AR degrader and directly compared its effect with those of the AR degraders, ARV‐110 and ARCC‐4. UBX‐390 showed prolonged AR degradation and inhibition of AR signaling, suggesting that it is a possible therapeutic option to treat patients with CRPC who are resistant to chemotherapy.

## 5 Experimental Section

### Cell Culture

Both T47D and DU145 cells, purchased from the Korean Cell Line Bank, were grown in Roswell Park Memorial Institute‐1640 medium (RPMI) (HyClone Laboratories Inc., Logan, UT, USA) supplemented with penicillin (100 U mL^−1^), streptomycin (100 µg mL^−1^), and 10% fetal bovine serum (FBS) (HyClone). LNCaP cells, purchased from the Korean Cell Line Bank, were grown in the same RPMI medium but with the addition of 25 mm *N*‐2‐hydroxyethylpiperazine‐*N*‐2′‐ethanesulfonic acid buffer. VCaP cells were grown in Iscove's modified Dulbecco's medium (HyClone) supplemented with penicillin (100 U mL^−1^), streptomycin (100 µg mL^−1^), and 20% FBS. 22Rv1 and HepG2 cells were grown in RPMI (HyClone) and DMEM (Hyclone) respectively, supplemented with penicillin (100 U mL^−1^), streptomycin (100 µg mL^−1^), and 10% FBS. HEK293 cells, purchased from the Korean Cell Line Bank, were grown in Minimum Essential Medium Eagle (MEM) (Sigma‐Aldrich, St. Louis, MO, USA) supplemented with penicillin (100 U mL^−1^), streptomycin (100 µg mL^−1^), and 10% FBS. Human PBMC cell stock (PBMNC050C; StemExpress, Folsom, CA, USA) was thawed in RPMI with 10% heat inactivated FBS according to the manufacturer's instructions and used in assays in RPMI supplemented with 10% FBS, penicillin (100 U mL^−1^), streptomycin (100 µg mL^−1^), 2 mm (1X) GlutaMax, and IL‐2 (20 Unit).

### Chemicals and Antibodies

The chemicals used in this study were DHT (D‐073; Sigma‐Aldrich), IL‐2 (PHC0021, Gibco, Waltham, MA, USA), bortezomib (S1013; Selleckchem, Houston, TX, USA), MLN4924 (5.05477.0001; Millipore, Burlington, MA, USA), bafilomycin (19‐148; Millipore), pomalidomide (S1567; Selleckchem), and cycloheximide (C4859; Sigma–Aldrich).

Primary antibodies against the following proteins were used for immunoblotting: GR (A303‐490A) and ELL2 (A302505A) from Bethyl Laboratories (Montgomery, TX, USA); PR (sc‐810), VHL (sc‐17780), PCLAF (sc‐390515), and hemagglutinin‐tag (sc‐7392) from Santa Cruz Biotechnology (Dallas, TX, USA); ER (8644), AR (5153S), glyceraldehyde‐3‐phosphate dehydrogenase (2118S), c‐myc (5605S), NKX3.1 (83700S), ERG (97249S), lamin B (17416S), α‐tubulin (2125s), and vinculin (4650S) from Cell Signaling Technology (Danvers, MA, USA); CRBN (NBP1‐91810) from Novus Biologicals (Centennial, CO, USA); CRISP‐3 (AF2397) from R&D Systems (McKinley Place NE, MN, USA); KLK2 (ab152136), SCD1 (ab39969), and BNIP3 (ab109362) from Abcam (Cambridge, UK).

### Immunoblotting

Total lysates were separated by sodium dodecyl sulfate‐polyacrylamide gel electrophoresis and transferred to nitrocellulose membranes using a Trans‐Blot Turbo Transfer kit (Bio‐Rad Laboratories, Hercules, CA, USA). Membranes were blocked with 5% skim milk powder in tris(hydroxymethyl)aminomethane‐buffered saline and Tween‐20 (TBST) (25 mm Tris pH 7.5, 150 mм NaCl, and 0.2% Tween‐20) for 45 min, at room temperature (25 °C), and then incubated with primary antibodies (1:1000 dilution in blocking buffer) overnight at 4 °C. After washing with TBST, the membranes were incubated with anti‐mouse IgG (7076; Cell Signaling Technology) or anti‐rabbit IgG (7074; Cell Signaling Technology) (1:5000 dilution in blocking buffer) for 45 min at room temperature. The membranes were then washed with TBST and developed using ECL reagent (SuperSignal Chemiluminescent Substrate, Thermo Fisher Scientific, Waltham, MA, USA) for detection using the Odyssey Infrared Imaging System (LI‐COR Biosciences, Lincoln, NE, USA).

### Immunofluorescence

VCaP cells seeded in an 8‐well chamber (154534PK; Thermo Fisher Scientific) were fixed with 4% paraformaldehyde and permeabilized with 0.1% Triton X‐100 (X100; Sigma‐Aldrich). After washing with phosphate‐buffered saline (PBS), the cells were blocked with 3% bovine serum albumin/PBS and incubated overnight at 4 °C with AR (rabbit; 1:50; 5153S; Cell Signaling Technology) and CRBN (mouse; 1:50; MAB9574‐10; R&D Systems) antibodies diluted in 3% bovine serum albumin/PBS. The following day, the cells were incubated in the dark with Alexa Fluor Plus 488 (mouse; 1:100; A11029; Invitrogen Corporation, Massachusetts, USA) and Alexa Fluor 647 (rabbit; 1:200; A21245; Invitrogen) secondary antibodies for 45 min. The slides were then mounted with Fluoro‐Gel II Mounting Medium (17985‐50; Electron Microscopy Sciences, Pennsylvania, USA) to visualize the nuclei. AR, CRBN, and nuclear staining were examined using a Zeiss LSM900 microscope (Carl Zeiss AG, Oberkochen, Germany).

### Cell Viability Assay

Cells seeded in white 96‐well microplates at a density of 500–2000 cells per well were treated with an AR inhibitor or degrader after serial dilution and incubated for the indicated number of days. According to the manufacturer's protocols, the cell viability assay was performed using the Cell Titer‐Glo 2.0 Assay Kit (G9242; Promega, Madison, WI, USA). The plates were equilibrated at room temperature for 30 min before Cell Titer‐Glo buffer was added in equal volumes to the cell culture medium in each well. The plates were mixed for 2 min to induce cell lysis and then incubated for 10 min at room temperature to stabilize the luminescence signal. Luminescence signals were measured using a microplate reader (Synergy H1; BioTek Instruments, Winooski, VT, USA). Data were analyzed using Prism version 5 (GraphPad Software, San Diego, CA, USA).

### Stable Cell Line Generation

The knockout or control PX459 plasmid was co‐transfected with packaging and VSV‐G envelope plasmids into 293T cells. Lentiviral supernatants were collected 48 and 72 h after transfection and added to VCaP cells with polybrene (8 µg mL^−1^). Puromycin (2 µg mL^−1^) selection was performed for two weeks to generate VCaP control and CRBN‐KO CRISPR pool cell lines. pSpCas9(BB)−2A‐Puro (PX459) V2.0 was a gift from Feng Zhang (Addgene plasmid # 62988; http://n2t.net/addgene:62988; RRID: Addgene_62988).

CRBN sgRNA sequence: 5′ AAAATCCTGTTCTTCTCGAT 3′

### Transient Transfection

HEK293 cells were seeded into 6‐well plates and transfected with pcDNA3.1‐AR WT, W742C, T877A, L702H, F877L, H874Y, or M896V plasmids using Lipofectamine 3000 Reagent (L3000001; Invitrogen) according to the manufacturer's protocol, with modifications. For each transfection, 0.4 µg of plasmid and 0.8 µL of each transfection reagent were added to Opti‐MEM (250 µL; Gibco). The mixture was incubated at room temperature for 15 min before addition to the wells. After 24 h of incubation, the transfected cells were seeded onto a 12‐well plate and treated with UBX‐390, enzalutamide, ARCC‐4, or ARV‐110 with cycloheximide (20 µg mL^−1^) for 24 h. The pcDNA3.1‐AR WT, W742C, and T877A vectors were gifts from Duke University, and other mutant vectors were generated by site‐directed mutagenesis of pcDNA3.1‐AR WT by Cosmo Genetech (Seoul, Republic of Korea).

### Nuclear Fractionation

Cells were collected in 50 mL tubes and centrifuged at 300 × *g* at 4 °C for 3 min. After resuspension in PBS (1 mL), 150 µL was transferred to a 1.5 mL microtube to obtain total cell lysates, while the remaining volume was transferred to another microtube for nuclear fractionation and centrifuged at 500 × *g* and 4 °C for 5 min. Total cell lysates were prepared in the same manner as that described for western blotting. For nuclear fractionation, each sample was centrifuged at 500 × *g* and 4 °C for 5 min, and the cell pellet was resuspended in cold hypotonic buffer (200 µL; 20 mm Tris‐HCl pH 7.4, 10 mm KCl, 2 mm MgCl_2_ solution, 1 mm EGTA, 0.5 mm DTT, and 0.5 mm PMSF with protease inhibitor) for swelling. NP40 was added at a final concentration of 0.1%, incubated for 3 min on ice, and centrifuged at 1000 × *g* at 4 °C for 5 min to separate the nuclei (pellet) and cytoplasm (supernatant). Cytosolic fractions were prepared by centrifugation at 15000 × *g* at 4 °C for 3 min, and the supernatant was transferred to a new tube on ice. Cell pellets (nuclei) were resuspended in isotonic buffer (1 mL; 20 mm Tris‐HCl pH 7.4, 150 mm KCl, 2 mm MgCl_2_, 1 mm EGTA, 0.5 mm DTT, and 0.5 mm PMSF) and incubated on ice for 10 min. After centrifugation at 1000 × *g* at 4 °C for 3 min, the supernatant was removed, and the remaining pellets were lysed with 30 µL RIPA buffer (RC2038‐050; Biosesang, Gyeonggi‐do, Korea). After centrifugation at 2000 × *g* and 4 °C for 3 min, nuclear fractions were prepared by transferring the supernatant to new microtubes. Fractionation was confirmed by western blotting.

### Proteomics Analysis

Cell lysates were digested using S‐Trap mini (C02‐mini‐80; ProtiFi, Fairport, NY, USA) according to the manufacturer's protocol version 4.7, which involves the use of tris(2‐carboxyethyl) phosphine and methyl methanesulfonate for reduction and alkylation, respectively. Trypsin/lysC (1 µg; A40009; Pierce Biotechnology, Union Grove, WI, USA) was added to each sample and incubated for 1 h at 47 °C. The eluted samples were dried using SpeedVac. The peptides were labeled with TMT10plex (90111; Thermo Fisher Scientific) following the manufacturer's protocol. After 1 h, the labeled peptides were mixed and desalted using Sep‐Pak (1 cc, 50 mg; WAT054955; Waters, Milford, MA, USA).

The mixed peptides were dried and dissolved in 5% acetonitrile and 10 mm ammonium bicarbonate solution. An Acquity UPLC system (Waters) equipped with a BEH C18 column (1.7 µm, 2.1 × 100 mm; 1860 02 352) and fraction collector (FC203B; Gilson, Middleton, WI, USA) was used for fractionation. Buffers A and B contained 10 mm ammonium bicarbonate in distilled water and 90% acetonitrile, respectively. The gradient was set from 5% B to 40% B for 79 min and from 40% B to 60% B for 16 min at a flow rate of 0.2 mL min^−1^. The eluted solution was collected after 0.8 min. Every 24th elution was merged to give 24 sub‐samples, such as e1, e25, e49, e73, e97‐frac1, e2, e26, e50, e74, and e98‐frac2. The samples were then dried using SpeedVac (Thermo Fisher Scientific).

The Ultimate RSLCnano 3000/Orbitrap Exploris 240 (Thermo Fisher Scientific) system was used for the analysis. Each sample was dissolved in 0.1% formic acid/5% acetonitrile (ACN) and peptides (0.5–1 µg) were loaded onto a trap column (PN 164535; Acclaim PepMap 100, 75 µm × 2 cm, C18, 3 µm). The peptides were separated using an analytical column (PN ES802; PepMap RSLC C18, 75 µm × 25 cm, 2 µm, 100 Å). The column temperature was set at 50 °C. The mobile phases used were 0.1% formic acid in water (Buffer A) and 0.1% formic acid in ACN (Buffer B). The following gradient was used at a flow rate of 300 nL min^−1^: 5% B to 7% B for 3 min, 7% B to 18% B for 73 min, 18% B to 25% B for 36 min, and 25% B to 60% B for 8 min.

The survey scan settings used were as follows: resolution = 120 000, maximum IT = auto, AGC = 300%, and mass range = 400–1600 Th. The selected precursor was fragmented by high‐energy collisional dissociation and analyzed using an Orbitrap. The tandem mass spectroscopy scan was performed using the following parameters: Top15 double play, 60 000 resolution, max IT of 200 ms, threshold at 5E3, normalized collision energy of 36%, isolation width of 0.4, dynamic exclusion after one occurrence, exclusion duration of 45 s, and a mass tolerance range of 10 ppm.

Raw data from liquid chromatography with tandem mass spectroscopy were analyzed using MaxQuant version 1.6.10.43 (Max‐Planck‐Institute of Biochemistry, Planegg, Germany). The key parameters were set as follows: database = UniProt *Homo sapiens*, enzyme = trypsin/P, variable modification = oxidation (M), acetyl (protein N‐term), fixed modification = methylthio (C), and type = reporter ion MS2 TMT10plex. Among the different result files, the “proteinGroups.txt” file was used for subsequent statistical computations using the Perseus version 1.5.8. Contaminant and reverse protein groups were excluded after file screening. Protein groups were chosen to ensure that each experimental group contained a minimum of two valid values. Any missing values were substituted for normally distributed values. The *p*‐value was then calculated for two of the three experimental groups. Significant protein groups were selected based on fold changes and *p*‐values.

### RNA‐Seq Library Construction

Total RNA was extracted using QIAzol (79 306; QIAGEN, Hilden, Germany), according to the manufacturer's instructions. RNA‐seq libraries were constructed using purified RNA (5 µg) and a NEXTflex Rapid Directional mRNA‐seq kit (NOVA‐5138‐11; PerkinElmer Informatics, Waltham, MA, USA). Briefly, mRNA was selected using poly(A) beads and fragmented using fragmentation enzymes. After the first‐ and second‐strand cDNA synthesis from a template of fragmented RNA, adenylation, and PCR amplification steps were performed according to the RNA‐seq library construction steps.

### Chromatin Immunoprecipitation (ChIP) and ChIP‐Seq Library Construction

The ChIP assay was performed as previously described.^[^ ^39^ ^]^ Briefly, 30 million VCaP cells were cross‐linked with 1% formaldehyde for 15 min, quenched with 0.125 m glycine for 10 min, and incubated for 10 min at 4 °C with 1 mL of cell lysis buffer containing protease inhibitor (cOmplete Protease Inhibitor Cocktail, 11 873 580 001; Roche Group, Basel, Switzerland) and 1 mm DTT (BP172; Fisher Bioreagents, Pittsburgh, PA, USA). After centrifugation at 5000 × *g* for 30 s, the isolated nuclei were gently resuspended in nuclear lysis buffer (1 mL) containing protease inhibitor and 1 mm DTT. The chromatin lysate was sonicated for 10 cycles (30 s on,30 s off) and centrifuged at 12000 × *g* for 15 min at 4 °C. The supernatant was pre‐cleared by incubation with 5 mL of immunoprecipitation dilution buffer, rabbit IgG (50 µg; I8140; Sigma), and Protein A magnetic beads (50 µL; 10002D; Invitrogen) for 1 h at 4 °C. Immunoprecipitation was conducted overnight in a rotator maintained at 4 °C with pre‐cleared chromatin (6 mL), AR antibody (5 µg; ab108341; Abcam), and Protein A magnetic beads (50 µL). The next day, the immunocomplexes were washed six times and eluted with elution buffer (200 µL) at 1000 rpm for 60 min in a thermomixer maintained at 45 °C. The eluate was de‐crosslinked with 0.25 м NaCl and incubated overnight in a water bath maintained at 65 °C. The next day, Proteinase K (4 µL; P8107S; New England Biolabs, Boston, MA, USA) was added and incubated for 2 h at 42 °C, following which the immunoprecipitated DNA was purified with a QIAquick PCR Purification Kit (28 106; Qiagen) in elution buffer (50 µL).

For ChIP‐seq library construction, the study used purified ChIP DNA (40 µL) and a NEXTflex ChIP‐seq kit (NOVA‐5143‐02; PerkinElmer). Briefly, ChIP DNA was end‐repaired, followed by A‐tailing and size selection (250–300 bp) using AMPure XP beads (A63881; Beckman Coulter, Pasadena, CA, USA). Other procedures, from adenylation to PCR amplification, were performed according to the ChIP‐seq library construction steps. Fifteen PCR cycles were used for the final library amplification, and the quality of the ChIP‐seq libraries was determined using a Bioanalyzer with a high‐sensitivity chip (Agilent Technologies, Santa Clara, CA, USA). ChIP‐seq libraries were sequenced using the Illumina NextSeq platform (Illumina, San Diego, CA, USA), with single‐end reads of 76 bases (LAS).

### Bioinformatics Analyses of ChIP‐Seq and RNA‐Seq

The RNA‐seq data were aligned by mapping the raw reads to the reference human genome assemblies (hg19) using STAR. Cufflink tools were used to analyze differentially expressed genes. To normalize the RPKM values obtained as described above, Z‐scores were calculated for the expression of genes corresponding to each gene group and plotted. Z‐scores were calculated by subtracting the overall average gene abundance from the raw expression for each gene and dividing the result by the standard deviation of all measured counts across all samples. To conduct GO analysis on DHT early‐ and late‐response genes, a list of predefined genes was employed as the input for GO analysis using the AmiGO tool.

To align the ChIP‐seq data, raw reads were mapped to the reference human genome assembly hg19 using the Bowtie2. SAM tools were used to remove duplicate reads. To identify the AR peaks (*n* = 14213), the processed BAM files were analyzed, and ChIP‐seq tags were calculated using the findPeaks tool. AR ChIP‐seq counts were subsequently normalized using Z‐scores. For visualization using the University of California, Santa Cruz genome browser, a bigWig file was created using the MakeBigWig tool (HOMER Suite). A density plot was created using the annotatePeaks tool (HOMER suite) by centering the AR peaks with an extension of +/− 5,000 bp.

### LDH Assay

Cells were seeded in white 96‐well microplates at densities ranging from of 20 000–700 000 cells per well. Following serial dilution, cells were treated with an AR degrader or inhibitor along with DHT, and then incubated for the specified number of days. The LDH assay was performed using the Cytotoxicity Detection Kit (LDH) (11 644 793 001; Roche), following the manufacturer's protocol. The plates were equilibrated at room temperature for 30 min and centrifuged at 250 × g, for 10 min at room temperature. The supernatant was transferred to a new 96‐well v‐bottom plate, centrifuged again at 250 × g, for 10 min at room temperature, and then transferred once more to a new 96‐well v‐bottom plate before adding the LDH assay mixture at equal volumes to the cell culture medium in each well. The plates were incubated for 30 min at room temperature in dark. The absorbance was measured at 490 and 620 nm using a microplate reader (Synergy H1; BioTek Instruments, Winooski, VT, USA) and cytotoxicity [%] was calculated using the equation provided in the manufacturer's instruction. Data were analyzed using Prism version 5 (GraphPad Software, San Diego, CA, USA).

### In Vivo Xenograft Model

CB17/SCID male mice were obtained from CLEA Japan (Tokyo, Japan). VCaP cells were subcutaneously injected into 6‐week‐old mice at a dose of 3 × 10^6^ cells per mouse using Matrigel (356 237; Corning Life Sciences, Tewksbury, MA, USA). When the tumors reached an average volume of 400–450 mm^3^, the mice were divided into three groups based on tumor size (*n* = 7). The vehicle control, UBX‐390 (10 mg kg^−1^), and enzalutamide (20 mg kg^−1^) were administered orally once daily for 14 d. Tumor size and body weight were measured thrice a week using calipers (CD‐15APX; Mitutoyo Corporation, Kanagawa, Japan), and the last tumor size was measured 20 h after the last administration before sacrifice. Tumor volume was calculated using the following formula: length × width^2^ × 0.5. All animal experiments were conducted in accordance with the guidelines approved by the Institutional Animal Care and Use Committee at Ubix Therapeutics (UBIX2019001; Seoul, Republic of Korea).

### Statistical Analysis

Immunoblotting experiments were conducted independently two times. The remaining experiments were conducted in duplicate. Statistical analyses for in vivo experiments included one‐way analysis of variance (ANOVA) tests (*n = 7*), while for in vitro experiments, Student's *t*‐tests were conducted using GraphPad PRISM 5 (GraphPad Software, San Diego, CA, USA). P‐values below 0.05 were considered significant in the following manner: ^*^ *p* < 0.05, ^**^ *p* < 0.01, ^***^ *p* < 0.001, and ^****^ *p* < 0.0001. Within graphs except RNA‐seq and ChIP‐seq analysis, lines or bars represent mean values while error bars indicate the SE of the mean. Within graphs of RNA‐seq and ChIP‐seq analysis, bars represent mean values ± SD of the mean. The expression levels of proteins in immunoblotting data were normalized to those of GAPDH, which served as a loading control. RPKM values and AR ChIP‐seq counts were subsequently normalized using Z‐scores.

## Conflict of Interest

S.H.L. and J.H.R. are shareholders in Ubix Therapeutics and have ownership interests (including stock, patents, etc.). Patent PCT/KR2021/0 09287 is held on UBX‐390. A patent application related to this work has been filed by Ubix Therapeutics, Inc. on behalf of the authors. S.L., Y.W., J.K., H.W.K., J.Y.P., B.S., Y.C., and J.A. are the employees of Ubix Therapeutics and have ownership interests in Ubix Therapeutics. The remaining authors declare no conflict of interest.

## Author Contributions

S.L. and H.‐R.K. contributed equally to this work. S.L., H.‐R.K., J.‐S.R., J.S., and S.H.L. conceptualized the study. S.L., H.‐R.K., Y.W., H.W.K., J.Y.P., B.S., Y.C., and J.A. performed the investigation. S.L. and H.‐R.K. visualized the data. J.H.R., J.‐S.R., J.S., and S.H.L. supervised the project. S.L. and H.‐R.K. drafted the original manuscript. J.K., J.‐S.R., J.S., and S.H.L. reviewed and edited the manuscript.

## Supporting information

Supporting Information

## Data Availability

The data that support the findings of this study are available from the corresponding author upon reasonable request.
