# Supplementary material for: UBX‐390: A Novel Androgen Receptor Degrader for Therapeutic Intervention in Prostate Cancer
Source: Adv Sci (Weinh). 2024 Jul 3;11(33):2400398. doi: 10.1002/advs.202400398 (PMC11434238; doi:10.1002/advs.202400398)
Supplement: Supplementary file 1 — Supporting Information [file ADVS-11-2400398-s001.docx]

Supporting Information

UBX-390: A Novel Androgen Receptor Degrader for Therapeutic Intervention in Prostate Cancer

Soohyun Lee, Hwa-Ryeon Kim, Yaejin Woo, Jiyoung Kim, Han Wool Kim, Ji Youn Park, Beomseon Suh, Yuri Choi, Jungmin Ahn, Je Ho Ryu, Jae-Seok Roe*, Jaewhan Song*, and Song Hee Lee*


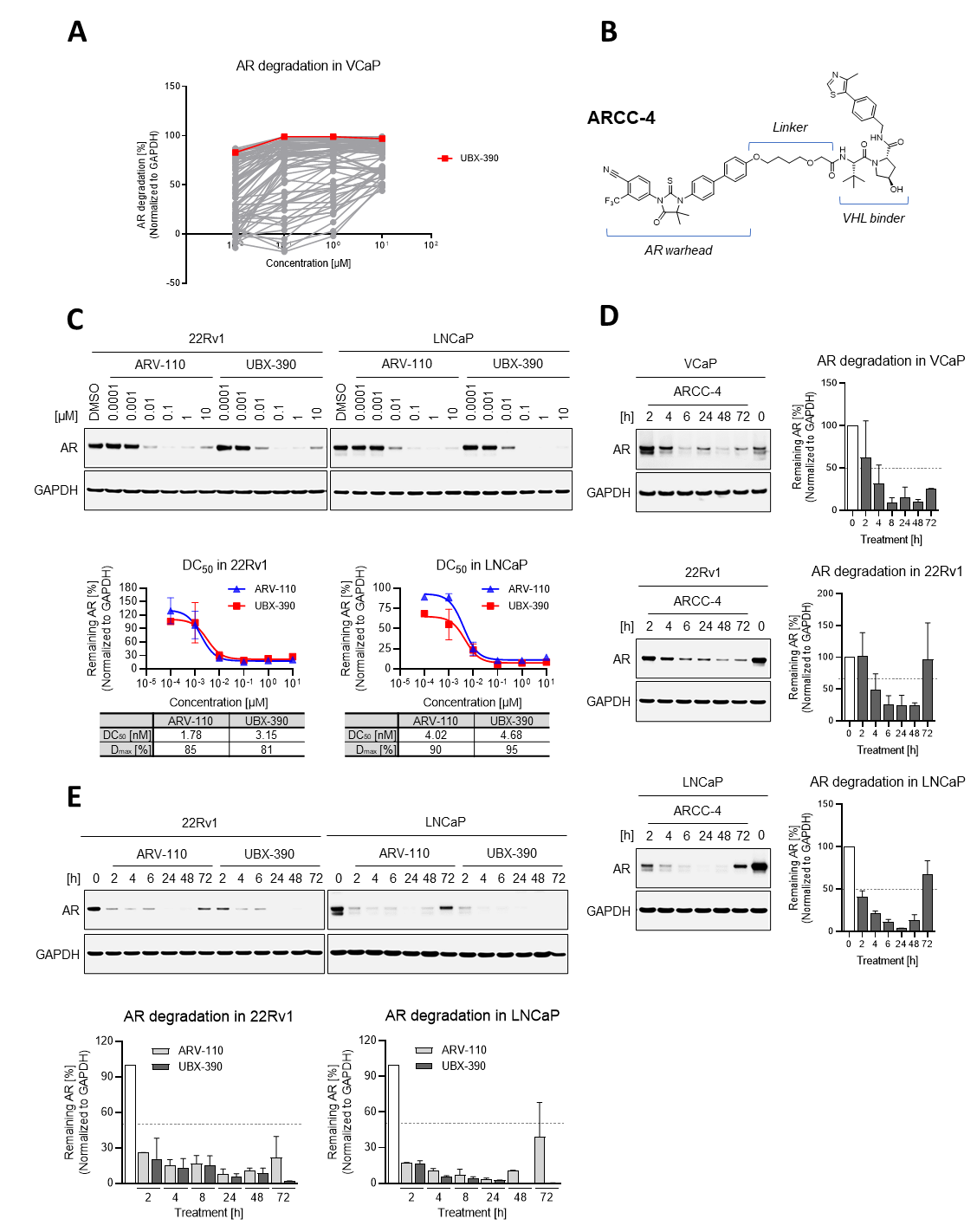


**Figure S1.** UBX-390 strongly induces AR degradation in prostate cancer cell lines. (**A**) Screening of 73 candidate AR degraders with different combinations of the linker and CRBN binder. (**B**) Chemical structure of ARCC-4. (**C**) Immunoblotting results depicting the AR-degradation effect of UBX-390 and ARV-110 in 22Rv1 and LNCaP cells after 20 h of treatment at different doses; (**D**) 0.1 μм ARCC-4 in VCaP cells at different time-points; and (**E**) 0.1 μм UBX-390, ARV-110, or ARCC-4 in 22Rv1 and LNCaP cells at different time-points. Immunoblotting experiments were conducted independently two times. The values for the remaining AR were normalized to those of GAPDH, which served as a loading control. Data are presented as mean values ± SE of the mean. GAPDH, glyceraldehyde-3-phosphate dehydrogenase; AR, androgen receptor.


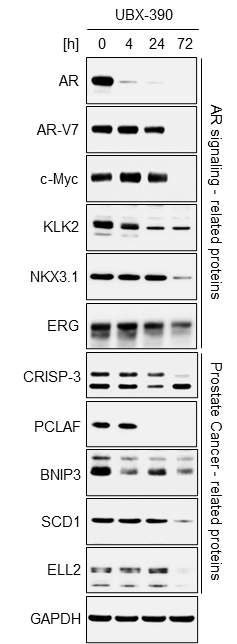


**Figure S2.** UBX-390 induces inhibition of AR signaling and prostate cancer-related pathway. Immunoblotting shows the effect of UBX-390 on AR signaling- and prostate cancer-related proteins in VCaP cells at different time-points. GAPDH, glyceraldehyde-3-phosphate dehydrogenase; AR, androgen receptor.


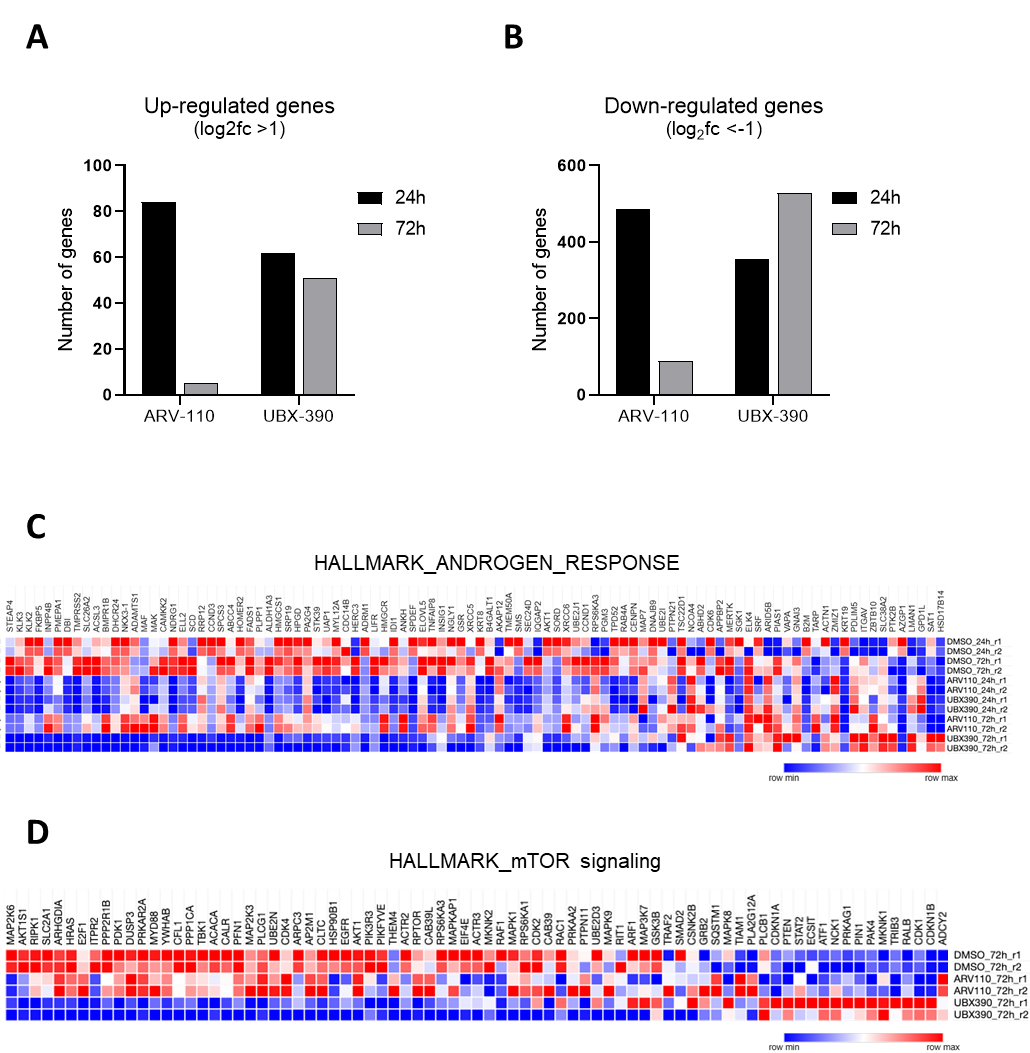


Figure S3. UBX-390 regulates AR-related genes in VCaP cells. The number of genes (A) up-regulated or (B) down-regulated by at least two-fold upon ARV-110 or UBX-390 treatment. (C) Heatmap showing the expression levels of androgen-response signature genes in VCaP cells following 24h or 72h treatment with ARV-110 or UBX-390. (D) Heatmap showing the expression levels of mTOR signaling signature genes in VCaP cells following 72h treatment with ARV-110 or UBX-390.

**
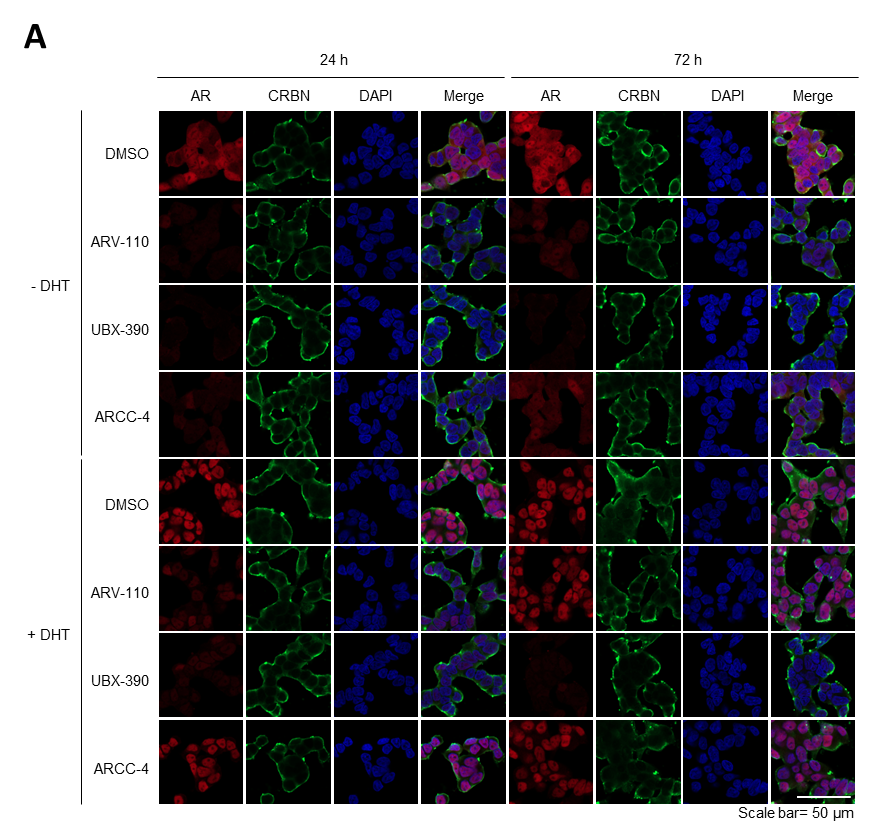
**

**Figure S4. UBX-390 induces AR degradation in both cytosol and nucleus.**

Images captured using confocal microscopy to compare the AR degradation effect of AR degraders in the presence of DHT after 24 or 72 h of treatment (400× magnification). Scale bar=50 µm. AR: androgen receptor; DHT: dihydrotestosterone.


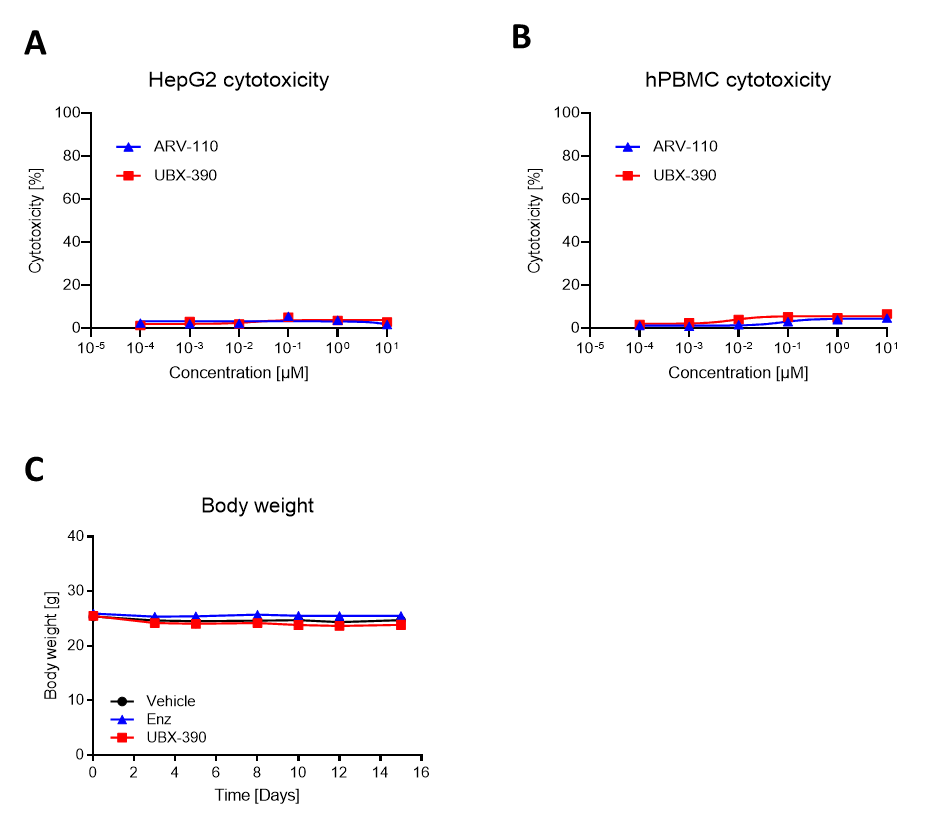


Figure S5. UBX-390 has no cytotoxicity *in vitro* and *in vivo.* Evaluation of cell cytotoxicity in (A) HepG2 cells; and (B) human PBMC after UBX-390 or ARV-110 treatment for 24 h. (C) Graph showing body weight following vehicle, UBX-390, or enzalutamide administration in the VCaP xenograft mouse models (*n*=7). The cytotoxicity experiments were conducted in duplicate. Data are presented as mean values ± SE of the mean. Enz, enzalutamide.


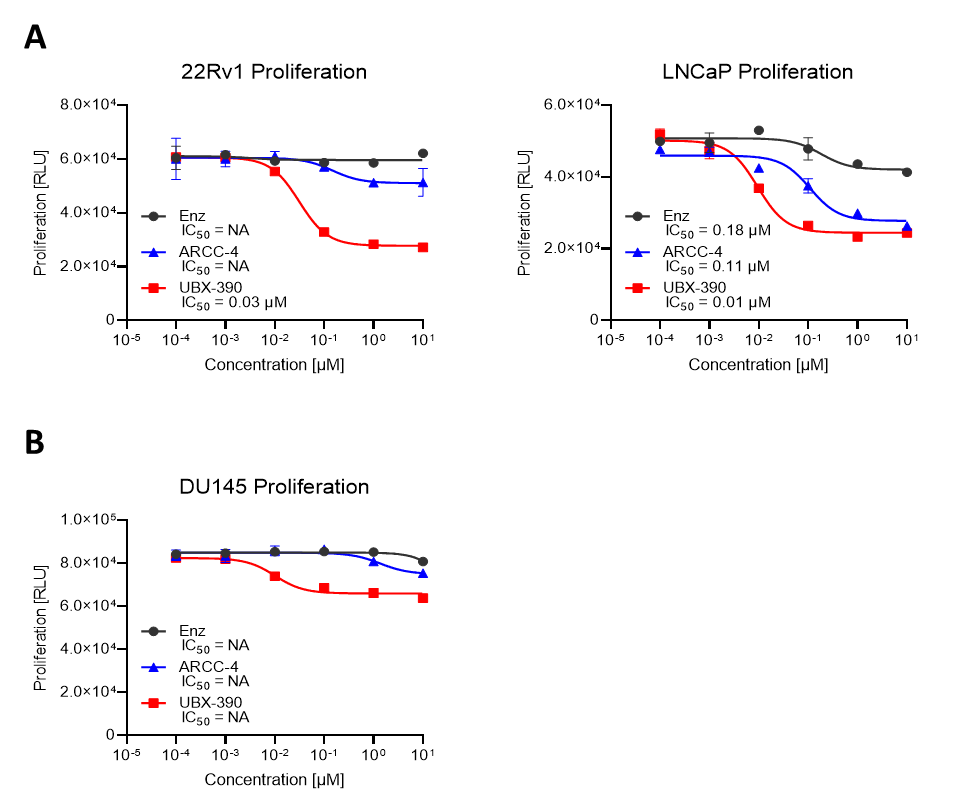


Figure S6. UBX-390 inhibits cell proliferation of AR-positive cell lines. Evaluation of the inhibitory effects of UBX-390, ARCC-4, and enzalutamide on cell proliferation in (A) 22Rv1, and (B) LNCaP and 22Rv1 cells, after treatment for 5 d; and (C) DU145 cells, after treatment for 4 d. The experiments were conducted in duplicate. Data are presented as mean values ± SE of the mean. NA, not applicable; Enz, enzalutamide.
